# The possible therapeutic role of curcumin and quercetin in the *early-stage* of COVID-19—Results from a pragmatic randomized clinical trial

**DOI:** 10.3389/fnut.2022.1023997

**Published:** 2023-01-18

**Authors:** Ikram Din Ujjan, Saeed Khan, Roohi Nigar, Hammad Ahmed, Sagheer Ahmad, Amjad Khan

**Affiliations:** ^1^Department of Pathology, Liaquat University of Medical and Health Sciences (LUMHS), Jamshoro, Pakistan; ^2^Department of Molecular Pathology, Dow University of Health Sciences, Karachi, Pakistan; ^3^Department of Obstetrics & Gynecology, Bilawal Medical College, LUMHS, Jamshoro, Pakistan; ^4^Bilawal Medical College, Jamshoro, Pakistan; ^5^Shifa College of Pharmaceutical Sciences, Shifa Tameer-e-Millat University, Islamabad, Pakistan; ^6^Nuffield Division of Clinical Laboratory Sciences, Radcliffe Department of Medicine, University of Oxford, Oxford, United Kingdom

**Keywords:** curcumin, quercetin, SARS-CoV-2, COVID-19, polyphenols

## Abstract

**Background:**

Curcumin (CUR) and quercetin (QUE), two natural polyphenols, possess diverse biological activities including broad-spectrum antiviral, antioxidant, and immunomodulatory effects. Both CUR and QUE have shown inhibition of severe acute respiratory syndrome coronavirus 2 (SARS-CoV-2) in *in vitro* assays.

**Objective:**

In the present study we aimed to assess the possible treatment benefits of a combined curcumin and quercetin (CUR-QUE) oral supplement, alongside standard of care (SOC), in the *early-stage* COVID-19 infection.

**Methods:**

This was an exploratory, pragmatic, open-label, randomized controlled clinical trial, conducted at the Department of Pathology, Liaquat University of Medical and Health Sciences, Jamshoro, PK. The study compared the treatment effect of an oral CUR-QUE supplement *plus* SOC vs. SOC alone, in the *early-stage*/mild to moderately symptomatic COVID-19 outpatients. Patients were randomized in a 1:1 ratio to CUR-QUE (*n* = 25) and control (*n* = 25) treatment groups. The CUR-QUE supplementation consisted of a daily intake of 168 mg curcumin and 260 mg quercetin, as two soft capsules, to be taken twice a day at home for 14 days.

**Results:**

After one-week of treatment, most of the patients in the CUR-QUE group showed an expedited clearance of the viral infection i.e., 18 (72.0%) vs. 6 (24.0%) patients in the control group tested negative for SARS-CoV-2 in the nasal-oropharyngeal swab reverse transcription-polymerase chain reaction (RT-PCR) analysis (*p* = 0.0002). In addition, COVID-19-associated acute symptoms were also speedily resolved in the CUR-QUE treated patients, i.e., 10 (40.0%) vs. 4 (16.0%) patients in the control group (*p* = 0.061). The CUR-QUE supplementation therapy was well-tolerated by all 25 patients and no treatment-emergent effects or serious adverse events were reported.

**Conclusion:**

The results revealed in this exploratory study suggest a possible therapeutic role of curcumin and quercetin in the *early-stage* of COVID-19. It is proposed that the two agents possibly acting in synergy, interfere the SARS-CoV-2 replication, and thus help a speedy recovery in the *early-stage* of COVID-19. Further research is highly encouraged.

**Clinical trial registration:**

Clinicaltrials.gov, Identifier NCT04603690.

## Introduction

Coronavirus disease 2019 (COVID-19) caused by the novel severe acute respiratory syndrome coronavirus 2 (SARS-CoV-2), has resulted in the death of over 6 million people worldwide. The COVID-19 pandemic is ongoing, affecting the communities and poses an enormous challenge to the healthcare systems. The mass vaccination has significantly controlled the worldwide SARS-CoV-2 infection, transmission, rate of hospitalization and mortality. Mutation in the virus poses a potential risk to the effectiveness of the COVID-19 vaccines. Low-/middle-income countries are still suffering from COVID-19 impacts due to many challenges such as lack of vaccine, funding, and COVID-19 medications.

The clinical manifestations of COVID-19 is rather heterogenous ranging from no symptoms to mild cough, pneumonia, acute respiratory distress syndrome (ARDS) and multi organ failure, with 10–20% of the symptomatic patients likely to develop serious illness (1). Host’s immune response is believed to be closely related with the severity and outcomes in patients with COVID-19 (2–5). Older age, male sex and comorbidities have been associated with worst outcomes in patients with COVID-19 (6–8). The clinical course of COVID-19 is unpredictable, which can rapidly change in an irreversible outcome. Amongst the unique characteristics of COVID-19 is a predilection to elicit a maladaptive immune response leading to an excessive systemic inflammation (cytokine storm) and organ injury (9, 10). Inflammation is particularly severe in the lungs and vascular endothelium and is mostly associated with substantial alveolar damage and thrombosis of large and small lung vessels (11). The so-called cytokine storm is believed to be the major underlaying cause of mortality in COVID-19 patients (9).

For the management of mild to moderate symptomatic COVID-19 infection, commonly used analgesics such as paracetamol and ibuprofen are advised as first-line treatment for most people to lower the temperature and treat body aches and pain. Several new COVID-19 antivirals including molnupiravir (12), sotrovimab (13), casirivimab/imdevimab (14), and nirmatrelvir/ritonavir (15) have been recently developed, and claimed to be effective to prevent hospitalization in patients who are at risk of developing severe COVID-19. But these drugs are highly costly and available only in certain developed countries such as the UK, and USA. For severe COVID-19 condition, anti-inflammatory intervention such as corticosteroids (dexamethasone) (16), interleukin-6 (IL-6) receptor blockers (tocilizumab) (17), and baricitinib (18), a Janus kinase inhibitor, have shown to result in clinical improvement and reduce mortality. The treatment benefits of these anti-inflammatory therapies are yet to be conclusively proven, and their pros and cons remain unknown. Though, it is very unlikely that a single magic bullet drug will cure COVID-19, but rather a combination of *early-stage* antiviral and anti-inflammatory agents will be the most effective therapy for COVID-19. Supressing the SARS-CoV-2 viral replication in the *early-stage* and concomitantly modulating the host’s hyperinflammatory response is believed to be crucial for early recovery and prevention from progression to severe illness. There is thus an urgent need of safe, effective, cheap, and worldwide available *early-stage* COVID-19 medications for speedy early recovery and control of COVID-19 community transmission.

Diverse nutritional agents with demonstrated antimicrobial, antioxidant and immunomodulatory (anti-inflammatory) activities are believed to act as an adjuvant in the body and improve the immune and antioxidant defense systems against pathogenic infections. Amongst such agents, curcumin (CUR) (19–21), and quercetin (QUE) (22–24), (hereafter combinedly referred to as CUR-QUE), two most extensively studied polyphenols, have been proposed as possible adjuvants for *early-stage* COVID-19 due to their demonstrated antiviral, antioxidant, and immunomodulatory pharmacological effects.

Curcumin is the main curcuminoid present in the roots of turmeric (*Curcuma longa*) spice, has shown diverse pharmacological activities such as antioxidant, anticancer, antibacterial, antiviral, antidiabetic, and anti-inflammatory, both in *in vitro* and animal model studies (25). Over 300 clinical trials have reported the beneficial protective effects of curcumin against various diseases including respiratory disease such as chronic obstructive pulmonary disease (COPD), asthma, pulmonary fibrosis, acute lung injury, inflammatory diseases such as inflammatory bowel disease (IBD), rheumatoid arthritis, psoriasis, neurological diseases, cardiovascular diseases, metabolic diseases, liver diseases, and cancers (26–28). Curcumin reportedly is also a broad-spectrum antiviral, inhibiting a variety of viruses such as SARS-CoV-1 (29), human immunodeficiency virus (HIV)-1, HIV2, herpes simplex virus (HSV), human papillomavirus (HPV), human T-lymphotropic virus-1 (HTLV1), hepatitis B virus (HBV), hepatitis C virus (HCV), influenza A virus, Japanese encephalitis virus (JEV) (30), and SARS-CoV-2 (31–34).

Quercetin, a flavonoid polyphenol, is found in the highest concentrations in citrus fruits, apples, red onions, grapes, dark cherries, and dark berries such as blueberries, blackberries and bilberries, green tea, buckwheat, parsley, sage, and olive oil. Quercetin exhibits diverse pharmacological effects such as anti-infective, anti-inflammatory, antibacterial, antioxidant, antiapoptotic, anticancer, and antidiabetic (35, 36). Quercetin also inhibits a variety of viruses, including SARS-CoV-1 (37, 38), Influenza virus, Ebola virus, HCV, HSV, Respiratory Syncytial viruses (39) and SARS-CoV-2 (31, 40, 41). In SARS-CoV-2-infected hamsters and mice, quercetin treatment alongside dasatinib has shown senolytic activity, resulting in reduction in the inflammation and improvement in pulmonary injury (42).

The demonstrated pharmacological activities and readily availability of curcumin and quercetin nutritional supplements prompted us to explore the treatment benefits of a combined curcumin and quercetin (CUR-QUE) oral supplement as an add-on to the standard of care (SOC) in the management of *early-stage*/mild to moderately symptomatic COVID-19 outpatients.

## Patients and methods

The study compared the treatment effect of a CUR-QUE supplement *plus* SOC vs. SOC alone, in the *early-stage*/mild to moderately symptomatic COVID-19 outpatients. This was a pragmatic, open-label, randomized controlled clinical trial conducted at the Department of Pathology, Liaquat University of Medical and Health Sciences (LUMHS), Jamshoro, Pakistan (PK). The study was carried out in accordance with the Declaration of Helsinki Ethical Principles and Good Clinical Practices (GCP), and approved by the Research Ethics Committee (REC), LUMHS *via* Ref. No. LUMHS/REC/137. Informed written consent was obtained from participants before enrolling in the study. The study has been registered at clinicaltrials.gov with registration number NCT04603690.

Patients were enrolled at the medical outpatients’ clinics of Liaquat Medical University Hospital, from 21 September 2021 to 21 January 2022. Study inclusion criteria include: male or female aged ≥18 years; confirmed SARS-CoV-2 infection as shown by nasal-oropharyngeal swab reverse-transcription polymerase chain reaction (RT-PCR)-based positive analysis, associated with mild to moderate typical acute COVID-19 acute symptoms such as fever, cough, myalgia, pharyngitis, asthenia, dysgeusia, dyspnea (SpO_2_ ≥ 93%, and not needing supplementary oxygen) etc., and can be treated as outpatients (do not require hospitalization). Exclusion criteria include: history of self-reporting hypersensitivity or allergic reaction to curcumin or quercetin, end stage kidney or liver disease, severe thrombocytopenia, or any other condition or factor that, in the opinion of the treating physician, contraindicates the use of CUR-QUE supplementation or makes the subject at risk due to their participation in the study.

### Randomization, treatment, and follow-up

**FIGURE 1:**
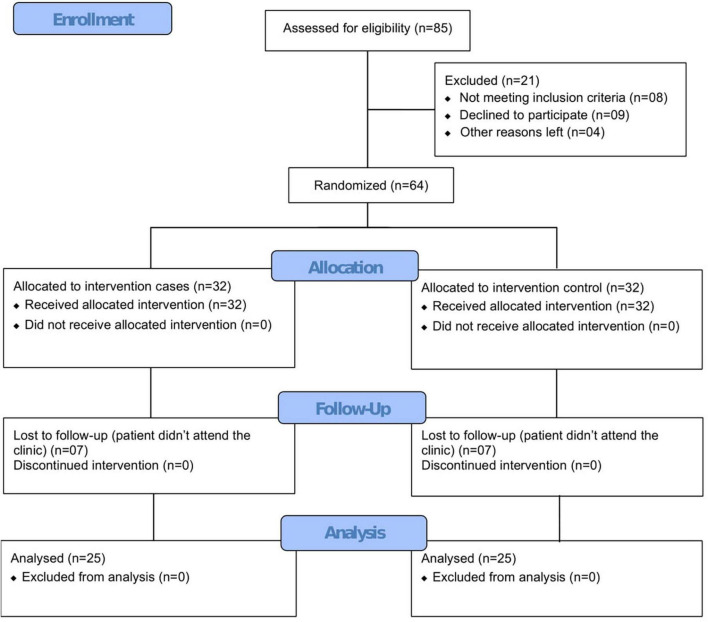
Study CONSORT flow diagram.

A total of 64 patients were enrolled and randomized in a 1:1 ratio to the CUR-QUE treatment arm (SOC *plus* CUR-QUE, *n* = 32) and control arm (SOC alone, *n* = 32). Randomization was performed by a trained healthcare professional who has no role in the study and carried out using a computer-generated random numbers code; patients assigned even numbers were allocated to the CUR-QUE treatment arm, and those assigned odd numbers were allocated to the control arm. Of the total 64 patients, 14 were lost in the follow-up, and the remaining 50 patients, consisting of 25 in each treatment arm (see CONSORT flow diagram in Figure 1), completed the study.

After treatment allocation, patient’s COVID-19-associated acute symptoms and serum levels of high-sensitivity C-reactive protein (hs-CRP), D-dimer, lactate dehydrogenase (LDH), and ferritin were evaluated and recorded by the outpatient’s physician, and treatment prescribed as per the randomization. Patients in both groups received the same SOC medications as per the hospital guidelines and include paracetamol 500 mg, oral azithromycin 500 mg, oral prednisolone 5 mg, with or without inj. ceftriaxone 1 g. The CUR-QUE supplementation to be taken at home, comprised of a daily intake of 168 mg curcumin, and 260 mg quercetin as two soft oral capsules, twice a day for 14 days. Each CUR-QUE supplement capsule (Nasafytol^®^, manufactured by Tilman SA, Belgium) contained 42 mg curcumin, and 65 mg quercetin, in a blend with 2.25 μg vitamin D3.

All patients were booked for day 7 follow-up in-person appointment with the physician at the medical outpatient’s clinic to test for the persistence of the SARS-CoV-2 infection (RT-PCR assay), assess COVID-19-associated acute symptoms, and evaluate laboratory biochemistry. If necessary, a further RT-PCR test was booked for day 14 of the treatment.

### Primary and secondary outcomes

The proportion of the patients testing negative for SARS-CoV-2 in the RT-PCR test, and improvement in the COVID-19-associated acute symptoms at day 7 follow-up, were the primary outcomes of this study, while improvement in the laboratory biochemistry was the secondary outcome of this study. Other outcomes include CUR-QUE supplement safety and tolerability, hospitalization rate and length, length of supplementary oxygen support, rate of intensive care unit (ICU) transfer and mortality.

### Statistical analysis

Welch two-sample *t*-test (normally distributed variables) was used to compare the continuous variables between the groups. Binary and categorical variables were described as counts (and%) and compared between groups using the Chi-squared test or, when counts were sparse, then with Fischer’s exact test. The *t*-tests and Chi-squared tests were performed using MedCalc for Windows, version 19.4 (MedCalc Software, Ostend, Belgium). Given the highly skewed distributions, non-parametric analysis of Covariance (ANCOVA) was used to test the effect of treatment on follow-up biomarker levels adjusted for their baseline values (43, 44) using the sm package in R 4.1.0 (45).

## Results

Patient’s demographics and baseline clinical characteristics are shown in Table 1. Overall, the median (IQR) age was 37.0 (48.0, 30.0) years and include 32 (64.0%) males. Of the total 50 patients, 17 (34.0%) had a history of underlying medical condition, including 6 (12.0%) patients had two or more conditions. The prevalent comorbidities were diabetes mellitus (DM), hypertension (HTN), and asthma in 9 (18.0%), 7 (14.0%), and 4 (8.0%) patients, respectively. At baseline, 26 (52.0%) patients presented five or more COVID-19-associated acute symptoms, while 24 (48.0%) patients had four or less symptoms. The most common symptoms were pyrexia [43 (86.0%)], cough [41 (82.0%)], myalgia [37 (74.0%)], pharyngitis [21 (42.0%)], flu [19 (38.0%)], and asthenia [27 (54.0%)]. Less common symptoms included dyspnea [17 (34.0%)], anosmia [18 (36.0%)], and dysgeusia [5 (10.0%)]. Most patients [48 (96.0%)] had received at least one dose of COVID-19 vaccination. Both the treatment groups were reasonably balanced with respect to the proportion of gender, age, COVID-19 vaccination status, and numbers of COVID-19-associated symptoms. The prevalence of myalgia and asthenia was higher in the control arm, while there were more patients with comorbidities in the CUR-QUE arm, incurred likely due to chance.

**TABLE 1:** Patients’ demographics and baseline clinical characteristics.

Baseline characteristics	CUR-QUE arm (*n* = 25)	Control arm (*n* = 25)	*p*-value
Age, median (IQR) (years)	38.0 (50.0, 35.0)	36.0 (41.0, 30.0)	–
Age, mean (SD) (years)	41.5 (13.7)	37.1 (9.4)	0.186
Sex, male, *n* (%)	15 (60.0%)	17 (68.0%)	0.557
**Comorbidity, (*n*%)**	14 (56.0%)	3 (12.0%)	0.001
HTN, *n* (%)	8 (32.0%)	1 (4.0%)	0.010
DM, *n* (%)	6 (24.0%)	1 (4.0%)	0.043
Asthma, *n* (%)	3 (12.0%)	1 (4.0%)	0.302
Others, *n* (%)	3 (12.0%)	1 (4.0%)	0.302
≥2 Comorbidities, *n* (%)	5 (20.0%)	1 (4.0%)	0.084
**COVID-19 symptoms**			
≥5 symptoms, *n* (%)	11 (44.0%)	15 (60.05)	0.262
≤4 symptoms, *n* (%)	14 (56.0%)	10 (40.0%)	0.262
Pyrexia, *n* (%)	21 (84.0%)	22 (88.0%)	0.686
Cough, *n* (%)	21 (84.0%)	20 (80.0%)	0.715
Myalgia, *n* (%)	13 (52.0%)	24 (96.0%)	0.0004
Pharyngitis, *n* (%)	12 (48.0%)	9 (36.0%)	0.394
Flu, *n* (%)	11 (44.0%)	8 (32.0%)	0.386
Asthenia, *n* (%)	9 (36.0%)	18 (72.0%)	0.011
Dyspnea, *n* (%)	9 (36.0%)	8 (32.0%)	0.767
Anosmia, *n* (%)	8 (32.0%)	10 (40.0%)	0.559
Dysgeusia, *n* (%)	5 (20.0%)	0 (0.0%)	0.019
COVID-19 vaccination status, *n* (%)	24 (96.0%)	24 (96.0%)	1.000

*n*, number of patients; IQR, interquartile range; SD, standard deviation; HTN, hypertension; DM, diabetes mellitus. *p*-values from *t*-tests and Chi-squared tests are shown for continuous and binary variables, respectively to highlight where randomization has not achieved balance between the two groups due to chance.

## Outcomes of the study

### Primary outcomes

**FIGURE 2:**
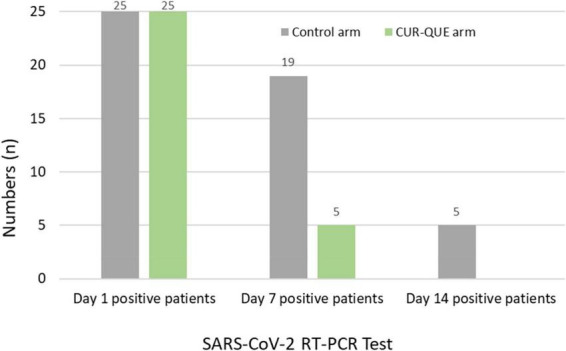
Patient’s RT-PCR COVID-19 test at day 1 and follow-up 7 and day 14 in the two treatment arms. Two patients in the CUR-QUE arm abstained the day 7 test.

Patients’ follow-up day 7 RT-PCR SARS-CoV-2 test results are shown in Figure 2. The results revealed that most of the patients in the CUR-QUE arm cleared the viral infection faster as compared to the control arm i.e., 18 (72%) of patients [including 9 (36%) patients with a history of underlying health condition] tested negative for SARS-CoV-2 vs. only 6 (24.0%) patients in the control arm; showing a statistically significant difference, *p* = 0.0002. Two patients in the CUR-QUE arm abstained COVID-19 test at day 7 follow-up appointment. Of the remaining 5 CUR-QUE and 19 control arm patients which were still SARS-CoV-2 positive on day 7, all of the CUR-QUE arm patients and 14 of the control arm patients tested negative at day 14, implying a delayed viral clearance in the control arm.

**FIGURE 3:**
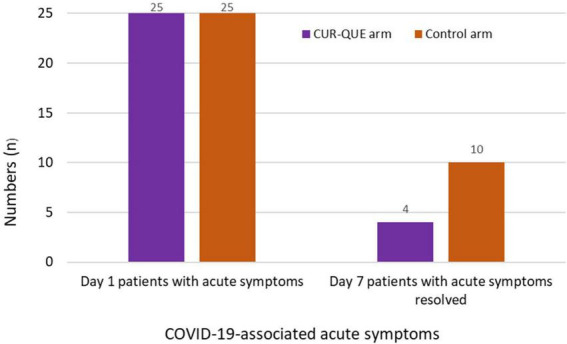
Patient’s COVID-19-associated symptoms at day 1 and follow-up day 7 in the two treatment arms.

In evaluating COVID-19-associated acute symptoms at follow-up day 7 (Figure 3), the number of patients showed improvement in the symptomatology was higher in CUR-QUE arm, i.e., 10 (40.0%) patients showed complete symptoms resolution, while 3 (12.0%) patient’s symptoms had reduced to three, 3 (12.0%) patients to two, and 9 (36.0%) patients symptoms reduced to one. In comparison, in the control arm, symptoms had resolved in only 4 (16.0%) patients, while 3 (12.0%) patients were left with four symptoms, 10 (40.0%) with three-symptoms, and 8 (32.0%) patients with two-symptoms. These results demonstrate a speedy recovery from COVID-19-associated acute symptoms in CUR-QUE arm as compared to control arm, which showed only partial symptoms improvement.

### Secondary outcome

Table 2 shows laboratory biochemistry (hs-CRP, D-dimer, LDH, ferritin) at baseline and day 7 follow-up by treatment group. Baseline serum levels of inflammatory biomarkers were comparable in the two groups. Both groups showed small change in D-dimer, LDH and ferritin levels between baseline and day 7 follow-up. Levels of hs-CRP fell in both groups, and there was weak evidence for a slightly greater fall in the CUR-QUE group, *p* = 0.068.

**TABLE 2:** Patients’ laboratory biochemistry at day 1 and follow-up day 7 in the two treatment groups.

	CUR-QUE arm		Control arm		*p*-value
**Biochemistry**	**Day 1**	**Day 7 follow-up**	**Day 1**	**Day 7 follow-up**	
hs-CRP median IQR (mg/L)^a^	3.5 (6.0, 1.0)	0.9 (1.2, 0.3)	2.6 (3.8, 2)	1.7 (3.6, 1.1)	0.068
D-dimer median IQR (mg/L)^b^	0.5 (0.7, 0.3)	0.2 (0.3, 0.1)	0.6 (0.9, 0.4)	0.3 (0.6, 0.1)	0.670
LDH median IQR (IU/L)^c^	314.0 (432, 246)	317.0 (412.7, 245.2)	344.0 (460.0, 290.0)	321.1 (390.0 282.1)	0.401
Ferritin median IQR (ng/ml)^d^	305.0 (580.0, 206.4)	298.1 (331.0, 223.7)	282.0 (345.4, 175.6)	250.3 (464.2, 150.3)	0.402

*p*-values for treatment effect on follow-up values adjusted for day 1 levels from non-parametric ANCOVA. Normal reference range; ^a^0.5–10 mg/L; ^b^up to 0.50 mg/L; ^c^140–280 IU/L; ^d^12–300 ng/ml (male), and 12–150 ng/ml (female).

### Other outcomes

Overall, in both the treatment groups patients showed recovery from COVID-19 and there was no case of hospitalization. The CUR-QUE supplementation was well-tolerated by all 25 patients, and no side-effects, or serious adverse events were reported.

## Discussion

In this exploratory pragmatic randomized clinical trial involving *early-stage*/mild to moderately symptomatic COVID-19 outpatients, those who received the CUR-QUE supplementation alongside SOC, showed a speedy recovery from COVID-19 as compared to patients who received the SOC alone. By treatment day 7, most of the patients in the CUR-QUE arm (i.e., 18 patients out of 25, 72.0%), including 9 (36.0%) patients with a history underlying health condition, cleared the SARS-CoV-2 infection earlier as compared to the control arm (i.e., 6 patients out of 25, 24.0%), showing a statistically significant difference (*p* = 0.0008). In addition, patients in CUR-QUE arm showed a speedy improvement in the COVID-19-associated acute symptoms as compared to the control arm i.e., 10 patients in the CUR-QUE arm showed complete symptoms resolution vs. 4 patients in the control arm (40.0% vs. 16.0%), *p* = 0.061. The results of this exploratory study suggest the possible adjuvant therapeutic effect of CUR-QUE nutritional supplement for *early-stage*/mild to moderate symptoms of COVID-19. It is speculated that the observed treatment benefits of CUR-QUE supplementation is possibly due to *synergistic* pharmacological effects of curcumin and quercetin. The speedy SARS-CoV-2 viral clearance in patients who received the CUR-QUE as shown in the RT-PCR analysis, is likely due to the interference in the replication of main protease (Mpro) of the SARS-CoV-2 by curcumin or quercetin or both as reported in the *in vitro* studies (31–34, 37, 40, 41). Mpro is a crucial enzyme involved in the replication of SARS-CoV-2 and is currently the inhibition target for the development of COVID-19 antiviral therapies. Other antiviral mechanisms shown by curcumin (19, 21) and quercetin (22, 24, 46, 47) including direct binding to the SARS-CoV-2 Spike protein (S-protein) RBD or disrupting the interaction of SARS-CoV-2 S-protein with its human specific receptor ACE2, which can prevent the viral entry to the host cells, are also possible.

The possible adjuvant effect of CUR-QUE supplementation alongside SOC in patients with COVID-19 have been investigated in several clinical trials. In the management of mild to moderately symptomatic COVID-19 patients, several trials have reported the possible adjuvant effect of oral curcumin supplementation, including speedy clearance of the SARS-CoV-2 in the RT-PCR test, early resolution of the COVID-19-associated acute symptoms, increase in oxygen saturation level, reduction in serum CRP levels, increase in lymphocyte count, reduction in duration of supplemental oxygen and hospitalization period (48–53). Several other clinical trials in hospitalized COVID-19 patients have also revealed the immunomodulatory (anti-inflammatory) effects of curcumin therapy, including reduction in the serum levels of IL-1β, IL-6, INF-γ, TNF-α, IL-17, IL-10, IL-35, TGF-β, PCT, FoxP3, IL-10, IL-35, TGF-β; reduction in the expression levels of IL-1β, IL-6, TBX21, and FOXP3, FoxP3, IL-10, IL-35, and TGF-β as well as upregulation of the frequency of Treg cells, reduction in the number of Th17 cells, Th17 cell-related cytokines levels, and downregulation of Th17 cell-related factors (54–59).

Similarly, the possible adjuvant effect of quercetin has also been investigated in several clinical trials in mild to moderately symptomatic COVID-19 patients, and have revealed beneficial effects including speedy viral clearance, early resolution of COVID-19-associated acute symptoms, and improvement in the serum levels of inflammatory biomarkers such as CRP, LDH, and alkaline phosphatase (ALP) (60–65). In study by Zupanets et al. in hospitalized COVID-19 patients associated with pneumonia, intravenous administration of quercetin/polyvinylpyrrolidone, followed by oral therapy of quercetin/pectin, showed accelerated restoration of the lung function (oxygen saturation, cough) and stabilization of the D-dimer level (66).

Moreover, the results observed in the present study are also in agreement and confirm the results of our previous collaborative study involving the same CUR-QUE supplement as an add-on to the SOC, in the management of mild to moderately symptomatic COVID-19 outpatients (67). Both studies showed a speedy viral clearance as shown in the RT-PCR test, and early resolution of the COVID-19-associated acute symptoms as compared to the SOC alone.

Both curcumin and quercetin supplementation have excellent safety profile, tolerability, and doses up to up to 8 g/day (68) and 1 g/day (69) respectively, for 3-months have failed to produce serious adverse effects. Both curcumin and quercetin have achieved the FDA GRASS (Generally Recognised As Safe) status for human use as nutritional supplements. The reported broad-spectrum antiviral, immunomodulatory, and antioxidant effects of curcumin and quercetin, and the results revealed in COVID-19 studies, supports the adjuvant therapeutic effect of these two polyphenols in the *early-stage*/mild to moderate symptoms of COVID-19. Supplementation of CUR-QUE in COVID-19 is suggested to have a greater impact when used *early* in the disease (i.e., outpatients) potentially preventing the spread of infection, hospitalization, and subsequent deaths, while reducing the healthcare system pressure, particularly in low-/middle income countries.

Limitations of our study include small sample-size and not been a double-blinded and placebo-controlled trial. Nevertheless, we have carried out a pragmatic randomized clinical trial that evaluated the treatment benefits of CUR-QUE supplement in patients with *early-stage*/mild to moderate symptoms of COVID-19, including in patients with underlying health conditions. Curcumin and quercetin supplements are safe, cheap, and worldwide available. The results revealed in this study are easily applicable in community-based patients and could possibly help in the management of mild to moderately symptomatic COVID-19 outpatients.

## Conclusion

According to the results of this study, a daily oral co-supplementation of 168 mg of curcumin and 260 mg of quercetin by mild to moderately symptomatic COVID-19 outpatients for 14 days alongside SOC could possibly help in the early clearance of the viral infection, and resolution of the COVID-19-associated acute symptoms. Further research is highly encouraged.

## Data availability statement

The original contributions presented in this study are included in the article, further inquiries can be directed to the corresponding author.

## Ethics statement

The study was approved by the Research Ethics Committee (REC), Liaquat University of Medical and Health Sciences (LUMHS), Jamshoro, Pakistan *via* Ref. No. LUMHS/REC/-137. The patients/participants provided their written informed consent to participate in this study.

## Author contributions

IU: study design, data collection, data interpretation, supervision, and critical revision of the manuscript. SK: study design, data interpretation, and manuscript revision. RN: data collection, supervision, and manuscript revision. HA: study administration, data collection, and manuscript revision. SA: study design, data interpretation, and critical revision of the manuscript. AK: study design, data interpretation, and writing the original draft and critical revision. All authors contributed to the article and approved the submitted version.
